# Identification of robust and generalizable biomarkers for microbiome-based stratification in lifestyle interventions

**DOI:** 10.1186/s40168-023-01604-z

**Published:** 2023-08-08

**Authors:** Jiarui Chen, Sara Leal Siliceo, Yueqiong Ni, Henrik B. Nielsen, Aimin Xu, Gianni Panagiotou

**Affiliations:** 1 Leibniz Institute for Natural Product Research and Infection Biology, Hans Knöll Institute -Microbiome Dynamics, Jena, Germany; 2 State Key Laboratory of Pharmaceutical Biotechnology, The University of Hong Kong, Hong Kong S.A.R., China; 3 Department of Medicine, The University of Hong Kong, Hong Kong S.A.R., China; 4 Clinical Microbiomics, Fruebjergvej 3, 2100 Copenhagen, Denmark; 5 Department of Pharmacology and Pharmacy, The University of Hong Kong, Hong Kong S.A.R., China; 6 Faculty of Biological Sciences, Friedrich Schiller University, Jena, Germany

**Keywords:** Gut microbiome, Microbiome dynamics, Resistance, Lifestyle intervention, Machine learning

## Abstract

**Background:**

A growing body of evidence suggests that the gut microbiota is strongly linked to general human health. Microbiome-directed interventions, such as diet and exercise, are acknowledged as a viable and achievable strategy for preventing disorders and improving human health. However, due to the significant inter-individual diversity of the gut microbiota between subjects, lifestyle recommendations are expected to have distinct and highly variable impacts to the microbiome structure.

**Results:**

Here, through a large-scale meta-analysis including 1448 shotgun metagenomics samples obtained longitudinally from 396 individuals during lifestyle studies, we revealed *Bacteroides stercoris*,* Prevotella copri*, and *Bacteroides vulgatus* as biomarkers of microbiota’s resistance to structural changes, and aromatic and non-aromatic amino acid biosynthesis as important regulator of microbiome dynamics. We established criteria for distinguishing between significant compositional changes from normal microbiota fluctuation and classified individuals based on their level of response. We further developed a machine learning model for predicting “responders” and “non-responders” independently of the type of intervention with an area under the curve of up to 0.86 in external validation cohorts of different ethnicities.

**Conclusions:**

We propose here that microbiome-based stratification is possible for identifying individuals with highly plastic or highly resistant microbial structures. Identifying subjects that will not respond to generalized lifestyle therapeutic interventions targeting the restructuring of gut microbiota is important to ensure that primary end-points of clinical studies are reached.

Video Abstract

**Supplementary Information:**

The online version contains supplementary material available at 10.1186/s40168-023-01604-z.

## Background

The human gut microbiome is a complex ecosystem made up of trillions of bacteria, viruses, archaea, and eukaryotic microbes contributing to essential functions in the host. Emerging studies have shown the close connection between the gut microbiome and human health and disease [1], such as influencing host nutrition and metabolism, training and modulating immune function, and contributing to patterns of brain development and behavior. Gut microbiota dysbiosis has been associated with several highly prevalent chronic diseases including gastrointestinal and neurological [2–4] disorders, metabolic diseases, as well as cardiovascular and respiratory illnesses [5–7]. Therefore, targeting the gut microbiome seems to be a promising strategy for restoring balance in the gut in order to improve the host’s health. However, unhealthy gut microbiota states can result in a recurring susceptibility to chronic illnesses and resistance to treatment efficacy [8].

Lifestyle interventions targeting the gut microbiota have been explored as a therapeutic treatment for numerous diseases. For example, prebiotic consumption, diet, and exercise have been associated with alterations in the gut microbiota structure and a positive impact on the host’s phenotype [9–11]. In most trials, large inter-individual differences in the treatment response have been observed [8], and some of these differences may depend on subject-specific microbiome response to the perturbation. In most cases, the microbiome response is currently unpredicted. Consequently, gut microbiota stability, resilience, and resistance are crucial ecological features [12]. Therefore, it is urgent to understand the potential mechanisms involved in gut microbiome resistance that may govern the response to perturbations and to determine whether lifestyle interventions can shape gut microbiota composition towards resilient healthy states.

In order to shed light on the resistance potential presented by an individual gut microbial ecosystem, we performed a large-scale meta-analysis of metagenomics samples obtained from longitudinal lifestyle interventions and compared the responses with no-intervention and antibiotic treatment studies. Groups of “responders” and “non-responders” were defined by their magnitude of taxonomic changes to a diverse set of lifestyle interventions and characterized by distinct gut microbiota compositions and functional profiles. From a clinical and translational perspective, the ability to predict microbiome resistance to perturbation offers significant advantages to further optimize disease therapies through microbiome-informed patients’ stratification and possibly restore plasticity in patients with resilient dysbiosis microbiomes.

## Results

### The extent of microbiome compositional changes depends on the environmental stimuli and varies between individuals

In order to elucidate the compositional and functional characteristics of the gut microbiome that may predict the personalized responses of the microbial communities to lifestyle, we collected metagenomic shotgun sequencing data from 10 studies covering 467 subjects sampled longitudinally (1590 total in total) (Table 1).

**Table 1:** Description of the study cohorts used in the meta-analysis

Study	Disease	Intervention	Intervention information	Duration (days)	Number subjects/samples
Mehta et al., 2018 [13]	Healthy	No intervention	-	-	140/560
Poyet et al., 2019 [14]	Healthy	No intervention	-	-	91/558
Palleja et al., 2018 [15]	Healthy	Antibiotics	Meropenem, Gentamicin, and Vancomycin	4	12/24
Raymond et al., 2015 [16]	Healthy	Antibiotics	Cefprozil	7	18/36
Willmann et al., 2019 [17]	Hematological-Oncological disease	Antibiotics	Ciprofloxacin	6	20/40
			Cotrimoxazole		21/42
Louis et al., 2016 [18]	Obesity	Exercise/Dietary	Multidisciplinary weight-loss program (OPTIFAST® 52, Nestlé Inc.): psychology, medicine, dietetics (very low-calorie diet), and exercise	84	14/28
Mardinoglu et al., 2018 [19]	Obesity with NAFLD	Dietary	Low-carbohydrate diet with increased protein content	14	10/20
Zhao et al., 2018 [20]	T2D	Dietary	High fiber diet composed of whole grains, traditional Chinese medicinal foods, and prebiotics	84	71/142
Ni et al., (in press) [21]	NAFLD	Dietary	Diet with high resistant starch type II content	120	50/100
Liu et al., 2020 [9]	Prediabetes	Exercise	Exercise activity 3 days/week as a combined aerobic and strength training program	84	20/40

These included 1118 samples from subjects that did not undergo intervention. This allowed us to set a “response threshold” to differentiate between microbiome changes that could simply be considered as natural fluctuation, and significant alterations following various interventions. We also retrieved five cohorts with lifestyle-based treatment (165 subjects, 330 samples), including a low-carbohydrate diet with increased protein content (I_LCD); a high-fiber diet (I_HFD); a highly resistant starch type II (HRS); a multidisciplinary weight-loss program (I_MWP); and an exercise training program (I_ETP) (Table 1). Moreover, the dataset contains four cohorts with different antibiotic treatments (71 subjects, 142 samples): a cocktail of meropenem, gentamicin, and vancomycin (referred to from now on as A_MER–GEN–VAN); cefprozil (A_CEF); ciprofloxacin (A_CIP); and cotrimoxazole (A_COT). Taxonomic and functional profiling was performed with all samples from different cohorts simultaneously after passing through the quality control. Intraclass correlation coefficient (ICC) is a measure of reliability or reproducibility that can be used to quantify the biological variability of the microbiome structure, previously used by Sinha et al. [22] to compute the microbiome temporal stability. The genus-level ICC was calculated for different estimates of alpha (Shannon, Simpson, and Chao1 Index) and beta diversity (using the top principal coordinates analysis (PCoA) scores based on Bray–Curtis dissimilarity, and unweighted or weighted UniFrac distances) for every cohort in our study (Table S1). ICCs range from 0 (no stability) to 1 (perfect stability), where values below 0.5 indicate poor microbiome stability and above 0.5, high microbiome stability [23].

**Fig. 1:**
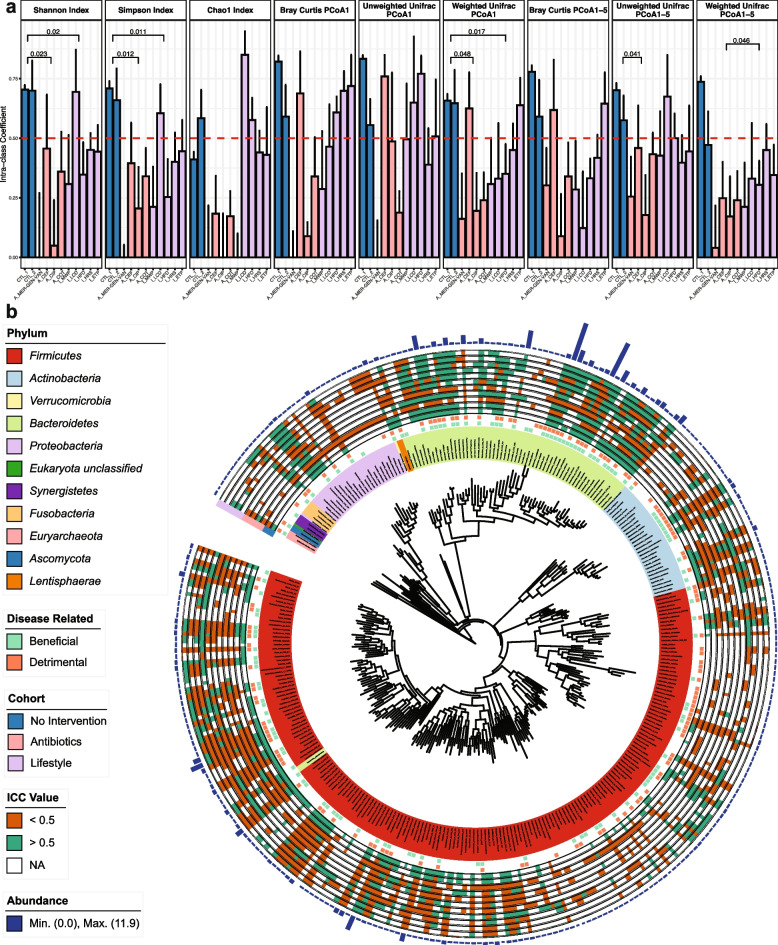
ICC evaluation of taxonomic profiles among study cohorts. **a** ICC values of alpha and beta diversity indexes at the species level in each study cohort. The error bars represent 95% confidence intervals. Cohort type is indicated by blue, pink, and lilac colors for no intervention, antibiotic intervention, and lifestyle intervention, respectively. Only significant *p* values are shown (Student *t* test, *p* < 0.05). The red dash line indicates an ICC value of 0.5. **b** Circos plot showing the annotated species in our metagenomics datasets in a phylogenetic tree. In the inner circles, disease-related species are shown in light green (beneficial) and light orange (detrimental). Species ICC values are indicated in orange (ICC < 0.5), green (ICC ≥ 0.5), and white (non-valid ICC). Barplots represent the median value of species abundance

We observed significantly higher mean ICCs values of Shannon and Simpson diversities for the two no-intervention cohorts (that did not include any intervention) compared to the four cohorts treated with antibiotics as well as the five cohorts with lifestyle interventions (Student *t* test, *p* < 0.05, Fig. 1a). The average ICC values of the two no-intervention cohorts remained high (> 0.50) for all diversity indexes, suggesting a stable gut microbiome alpha diversity in the absence of external disturbances. Interestingly, there is no significant difference in mean ICCs values of any alpha diversity index when comparing the four cohorts treated with antibiotics and the five cohorts undergoing lifestyle interventions (Student *t* test, *p* = 0.14, 0.240, 0.052 for Shannon index, Simpson index, and Chao1 index, respectively, Fig. 1a). Moreover, despite a clear trend of decreased ICCs for all beta diversity indexes in the 4 antibiotics cohorts compared to the two no-intervention cohorts, the result was not statistically significant (Student *t* test, *p* ≥ 0.05) except PCoA1 of Weighted Unifrac index and the average ICC value of PCoA1-5 of Unweighted Unifrac index. These results are probably due to the high variability observed between antibiotic types and personalized responses to each antibiotic. Nevertheless, the overall diversity ICC values of the cohort treated with a combination of meropenem, gentamicin, and vancomycin (A_MER–GEN–VAN) were extremely low with an average of 0.183, indicating a severe disturbance of the microbiome structure (Fig. 1a), while the ICC values of cohorts treated with cefprozil or cotrimoxazole were significantly higher than those of A_MER–GEN–VAN (paired *t*-test, adjusted *p* < 0.1) with an average of 0.453 and 0.368, respectively. On the contrary, the differences in the ICC values for beta diversity between no and lifestyle interventions were less obvious and again they were characterized by high variability among different types of intervention and of participants’ responses in each study group (Fig. 1a). By comparing the differences in the ICC values among the lifestyle intervention cohorts, we found that the I_MWP study, which used a multidisciplinary weight-loss program combining psychology, medicine, dietetics, and exercise, had average ICC values of 0.173 and 0.237, for alpha and beta diversity, respectively, significantly lower compared to all other single interventions (either dietetics or exercise) (paired *t*-test, adjusted *p* < 0.1). The comparisons among other cohorts in the lifestyle intervention category showed no significant differences (paired *t*-test, adjusted *p* ≥ 0.1). We further compared the beta diversity ICC values of the I_MWP with the four antibiotic-treated cohorts and interestingly, we found that its impact on microbial stability was higher than the A_CEF and A_COT studies (paired *t*-test, adjusted *p* < 0.1).

In summary, we generally observed that the microbial stability estimated as ICCs of alpha and beta diversity is disturbed by antibiotics and lifestyle interventions, but the extent depends on the specifics of each environmental stressor. Furthermore, the insignificance of beta dissimilarity between interventions and no intervention, which may be due to the variability of responsiveness among individuals, supports the notion that generic approaches to altering the microbiome structure in an unbalanced state may not bring the desired structural changes. The baseline microbiome could potentially define the magnitude of the response of the community structure to external stimuli, something that we further explored below.

### Lifestyle interventions could have a comparable impact with antibiotics on individual species' stability

Looking at the ICCs of the 309 individual species annotated in our metagenomics dataset, we observed a clear stratification among the no-intervention cohorts and the other two study groups (Fig. 1b). In the no-intervention cohorts, 79.6% of the detected species were regarded as stable (mean ICCs > 0.5), while this percentage dropped to an average of 27.3 and 43.6% for antibiotics and lifestyle intervention cohorts, respectively. When looking into the individual studies, we observed a similar tendency for the species stability as for the community diversity. The ICCs of 99% of the species in the A_MER–GEN–VAN study were < 0.5 indicating that almost all bacteria present in the microbial community were affected. The percentage of species having ICCs < 0.5 was high for all antibiotics (78.4, 62.3, and 50.3% for A_CIP, A_COT, and A_CEF, respectively). Interestingly, three of the lifestyle interventions had a similar or even higher impact than the administration of single antibiotics on the stability of individual species. The I_MWP intervention resulted in the highest percentage of species with ICCs < 0.5, affecting 71.6% of the community members. The studies using a high-fiber diet (I_HFD) and a high-resistant starch diet (I_HRS) were also characterized by a high percentage of species with ICCs < 0.5 (68.6 and 61.1%, respectively).

Looking for global taxonomic patterns in the lifestyle intervention cohorts, the statistical comparisons among the major phylum showed that the ICCs of *Bacteroidetes* species were significantly higher compared to *Firmicutes* and *Proteobacteria* species (Student *t* test, *p* < 0.05). Using the lifestyle intervention cohorts, we also examined whether the stability of species is correlated with their relative abundance at baseline. However, only 16 out of 309 species showed a significant correlation (Spearman correlation, adjusted *p* < 0.05) between the ICC and relative abundance. By extracting information from the Disbiome Database, we were able to retrieve disease associations for 162 species annotated in our metagenomics datasets. The stability of 115 out of the 162 disease-associated species could be influenced by at least one of the lifestyle interventions. The I_HFD study resulted in ICC values < 0.5 for 83 species associated with a wide range of metabolic diseases (obesity, type 2 diabetes, and hypertension) confirming the potential of a high-fiber diet as a way to target dysbiotic microbiome states. Disease-associated species, whose stability was uniquely influenced by particular lifestyle interventions, were also found. The I_HFD showed specificity towards 12 disease-associated species, whereas I_LCD, I_MWP, and I_HRS showed specificity towards 8, 6, and 3 species, respectively (Table S2).

In order to perform a comparative analysis among all interventions, we extracted 65 species with valid ICCs (not NULL value of ICCs) in every individual study. Out of the 65 species, 47 were stable (ICCs > 0.5) in the no-intervention cohorts; however, all of them lost their highly stable status in at least one study of either antibiotic treatment or lifestyle intervention. Interestingly, among these 47 species, we found 5 species, *Bacteroides massiliensis, Bacteroides stercoris, Barnesiella intestinihominis, Parabacteroides merdae,* and *Parasutterella excrementihominis, *that remained stable in all the lifestyle interventions (ICCs > 0.5). These 5 species further showed resistance (ICCs > 0.5) to two of the antibiotic treatments, cefprozil (A_CEF) and cotrimoxazole (A_COT), suggesting that these species are highly stable. The aforementioned 5 species have a conditional effect on human metabolic diseases, playing either beneficial (non-alcoholic fatty liver disease, cirrhosis, multiple sclerosis, etc.) or detrimental (autism, Parkinson’s disease, colon polyps, etc.) roles (Fig. 1b) [24]. We further explored the relative abundance of these 5 species across all samples and observed that they were all low-abundant species (< 0.92%), further confirming that stability and abundance are not correlated. *Alistipes indistinctus*, a species that has been associated with hypertension and autism, also showed an interesting stability pattern. *A. indistinctus* had a high prevalence of 32% but a low abundance of 0.25% on average. *A. indistinctus* showed high stability (ICC > 0.5) not only in the no-intervention cohorts but also in the cohorts with antibiotic treatments (except where the subjects were administered a cocktail dose of antibiotics, A_MER–GEN–VAN). Interestingly, a low-carbohydrate diet (I_LCD) and exercise (I_ETP) could result in an ICC < 0.5 for *A. indistinctus*, suggesting the potential of using specific lifestyle interventions to target highly stable and disease-associated species.

In summary, by evaluating the ICC value of each species across studies, we have identified both species that are highly resistant to any lifestyle and antibiotics intervention and species whose stability pattern can only be affected by specific lifestyle interventions. Interestingly, we also observed that lifestyle interventions can reach similar or even higher capability to impact the stability of microbial species as single antibiotics administration, questioning the broad characterization of antibiotics treatment as a more intense intervention compared to lifestyle interventions.

### Identification of species associated with microbiome responsiveness

**Fig. 2:**
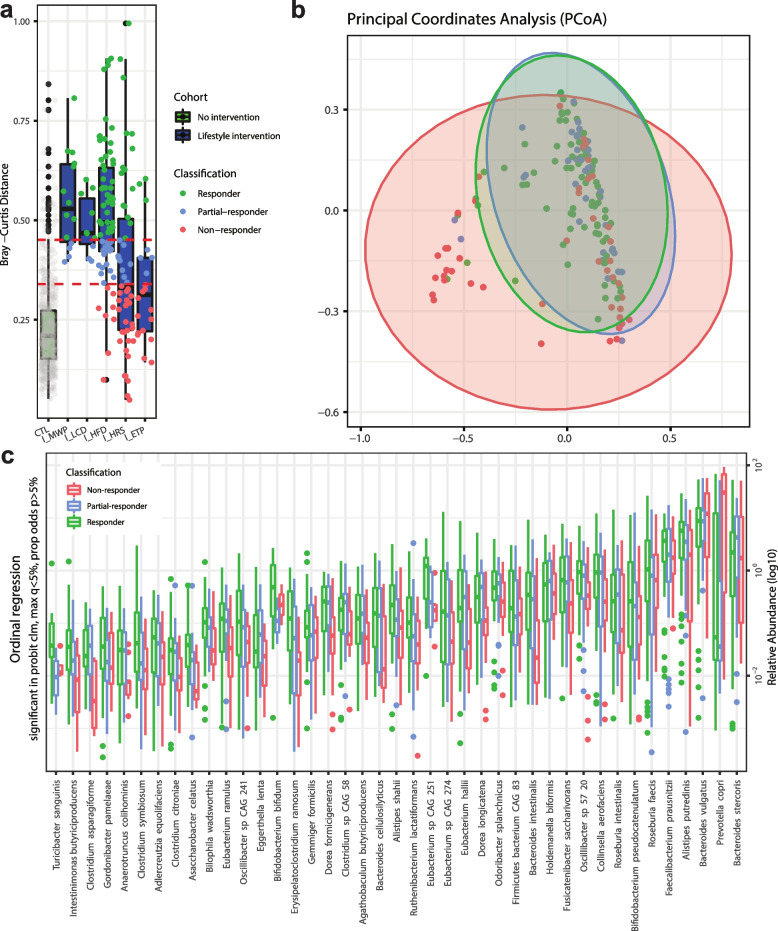
Microbiome compositional differences of responders, partial-responders, and non-responders to lifestyle interventions. **a** Bray–Curtis dissimilarity of longitudinal samples in subjects from no and lifestyle interventions. The two longitudinal no-intervention cohorts were combined in the first box. Bray–Curtis indexes dot colors indicate the microbiome response classification group by coral, blue, and green for non-responders, partial-responders, and responders, respectively. The two red dash lines represent the mean + SD and mean + 2*SD of the Bray–Curtis dissimilarities in the no-intervention cohorts as cutoffs to differentiate significant microbiome compositional changes from normal microbiome fluctuation. (CTL: study with no intervention; I_MWP: intervention study with multidisciplinary weight-loss program; I_LCD: intervention study with low-carbohydrate diet; I_HFD: intervention study with high-fiber diet; I_HRS: intervention study with high-resistant starch; I_ETP: intervention study with exercise training program). **b** Principal coordinate analysis of Bray–Curtis dissimilarity in non-responders, partial-responders, and responders to lifestyle interventions. **c** Relative abundances of the significant species using ordinal regression among non-responders, partial-responders, and responders groups (*p* < 0.05)

By calculating the day-to-day Bray–Curtis dissimilarities of each subject from the two longitudinal no-intervention cohorts, we established the criteria to differentiate effective response to a microbiome-targeted intervention from normal fluctuation of the microbial community composition. We used the mean + SD (68% population) and mean + 2*SD (95% population) of the Bray–Curtis dissimilarity (see “Methods” for details) as the two cutoffs to distinguish individual responses and formed the following groups for downstream analysis: (i) non-responders (< mean + SD), (ii) partial-responders ([mean + SD, mean + 2SD]), and (iii) responders (> mean + 2SD) as shown in Fig. 2a. By evaluating the dissimilarity before and after intervention of each subject among the five lifestyle intervention cohorts, 47.3% of individuals were classified as responders, while 24.2% were partial-responders, and the remaining 28.5% were grouped as non-responders. We calculated the species ICCs before and after intervention in each study and compared them based on the responder classification. The species ICCs were significantly lower in the responders compared to the non-responders (paired *t* test, *p* < 0.05), confirming the grouping.

We subsequently used the baseline microbiome samples of each subject to perform a principal coordinates analysis (PCoA) based on the Bray–Curtis dissimilarities. The microbiome composition of the subjects grouped by the newly constructed classification from non-responder to responder was significantly different (PERMANOVA, *p* < 0.05, R^2^ = 0.05, Fig. 2b). By comparing the species abundances between the non-responder, partial-responder, and responder groups, we found 41 species with significant differences among the groups (ordinal logistic regression, adjusted *p* < 0.2, Fig. 2c, Table S6). Interestingly, 37 out of the 41 species from the ordinal regression are highly stable species in the absence of interventions (ICCs > 0.5 in the no-intervention cohort), an important property for serving as biomarkers of community response. Among these 37 species, only 3 species were significantly enriched in the non-responder group, namely *Bacteroides stercoris*,* Prevotella copri*, and *Bacteroides vulgatus*. These 3 species remained significantly enriched in the non-responders group even when the lifestyle grouped subjects were combined with non-responders, partial-responders, and responders from the antibiotics cohorts. Similarly, 17 out of the 37 species were found significantly enriched in the responders group even when combining the lifestyle with the antibiotics cohorts, including *Collinsella aerofaciens*, *Gordonibacter pamelaeae*, *Ruthenibacterium lactatiformans*, *Turicibacter sanguini, Fusicatenibacter saccharivorans, Dorea longicatena,* and *Eubacterium hallii,* which were highly stable species (ICCs > 0.5).

#### Biosynthesis of amino acids and their taxonomic contributors as mediators of microbiome dynamic responses

Subsequently, we compared the MetaCyc pathway abundances among the three response groups using their baseline samples and found 116 pathways with significantly different abundances (Ordinal regression, adjusted *p* < 0.1), indicating a clear baseline stratification also at the functional level. Among the 116 pathways, enrichment of 34 was associated with non-responders and 82 with responders (Fig. S1). As observed with the species biomarkers of responsiveness, 97 out of the 116 pathways from the ordinal regression were highly stable in the absence of interventions (ICCs > 0.5 in the no-intervention cohort). We then investigated the contributions of the 41 significant species (Fig. 2c) to the 116 significant pathways using the stratified output of HUMAnN3. At least one of *B. stercoris, P. copri,* and *B. vulgatus*, the 3 species significantly enriched in non-responders, was taxonomically linked to 33 out of the 34 pathways enriched in non-responders, and all 3 species were contributing to the abundances of 24 pathways enriched in non-responders. Interestingly, when exploring the 82 pathways enriched in responders, we found only 4 pathways to have contributions from these non-responders associated species. Similarly, the 38 species enriched in responders were found to contribute to 51 out of the 82 pathways enriched in this response group.

**Fig. 3:**
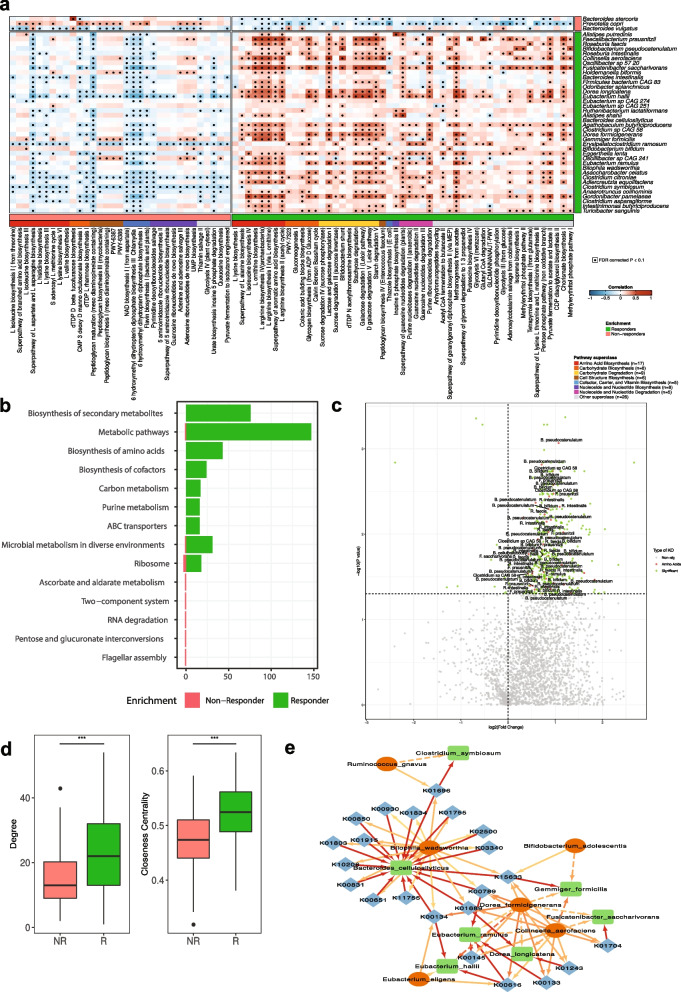
Microbiome functional differences of responders, partial-responders, and non-responders to lifestyle interventions. **a** Heatmap showing Spearman’s rank-based correlations between species and pathways with significantly different abundance (using ordinal regression among non-responders, partial-responders, and responders groups; adjusted *p* < 0.1). Only pathways with contributions from at least one of the species enriched in the same condition are shown. FDR-corrected *p* < 0.1 was deemed significant. The condition where the species or pathways are enriched is shown in coral and green for non-responders and responders, respectively. **b** Barplots showing the number of significant KOs mapped to each enriched pathway in responder and non-responder in green and coral, respectively. **c** Volcano plot of differentially abundant KOs based on the comparison between responders and non-responders. The log2 fold change and the log10 *p* values adjusted for multiple testing are plotted for each of the KOs. The dots marked with green represent significant KOs and the dots marked with red represent significant KOs involved in the biosynthesis of amino acids. The significant species which contributed to these KOs were annotated in the plot. **d** Comparison of the degree and closeness centrality between responders (R) and non-responders (NR) SparCC networks (Student *t* test, ***: *p* < 0.001). **e** Co-abundance network among species enriched in responders, the amino acid-related KOs, and amino acids (AA) auxotroph species (only significant correlations are considered, *p* < 0.05). Color intensity of the edges refers to the correlation value. The green, blue, and red color of the nodes represents species enriched in responders, the amino acid-related KOs, and AA auxotroph species, respectively

In order to further investigate the relationship between species, pathways, and microbiome response, we performed Spearman correlation analysis between the 41 significant species and the 84 significant pathways that they contributed to (Fig. 3a). A consistent pattern between the 3 species enriched in non-responders and specific functional groups was not observed, besides the significant positive correlations (Spearman, adjusted *p* < 0.1) with fucofuranose biosynthesis, flavin biosynthesis, and its precursors (Fig. 3a). On the contrary, a significantly larger and consistent pattern of positive associations between species and pathways was observed in the responders’ enriched taxonomic and functional signatures. Some of the strongest positive correlations were observed between the responders’ enriched species, including *C. aerofaciens*, *F. saccharivorans*, *E. hallii*,* Gemmiger formicilis*, and *G. pamelaeae*, and several pathways related to the biosynthesis of amino acids, e.g., arginine, isoleucine, and ornithine biosynthesis, among others (Fig. 3a). Metabolic cross-feeding of the aforementioned biosynthetically costly amino acids has been shown to promote stronger cooperative microbial interactions and drastically impact the community dynamics [25].

We subsequently performed differential abundance comparisons between responders and non-responders for the 2768 detected KEGG Orthology (KOs) and found 395 as significantly different (Wilcoxon rank sum test, adjusted *p* < 0.1). Among them, only 11 were more abundant in non-responders, whereas the remaining 384 KOs were highly abundant in responders (Fig. 3b). By mapping the significant KOs to the KEGG pathway database, the biosynthesis of secondary metabolites and biosynthesis of amino acids were two of the pathways with the highest KO contribution in responders, while very limited results were obtained for the non-responders (Fig. 3b). A significant enrichment of KOs related to the biosynthesis of amino acids in the responders compared to the non-responders was also observed (chi-square test, *p* < 0.01) with 43 KOs found to be significantly higher in responders, whereas none were higher in non-responders. Moreover, we investigated the species contributions to these amino acid-related KOs that were significantly enriched in the responders. We pinpointed 10 species that were top contributors to multiple significant KOs including *Faecalibacterium prausnitzii*,* Bacteroides cellulosilyticus*,* Fusicatenibacter saccharivorans*,* Eubacterium ramulus*, and *Eubacterium hallii* (Fig. 3c).

We subsequently built species co-abundance networks for responders and non-responders using the baseline samples, in order to further investigate the mechanisms by which responders’ enriched species regulate microbial community structural changes. We explored two commonly used centrality measures that reflect the flow of information in the network, the degree and closeness centrality. Responders have a more interconnected community network (Student *t* test, *P* < 0.001; Fig. 3d) and higher closeness centrality compared to non-responders (Student *t* test, *P* < 0.001; Fig. 3d). When the responder network was investigated in detail, we observed that 11 out of the 15 amino acid auxotroph (AAA) species identified recently in the study of Yu et al. [26] were present in the community network. These 11 AAA species had positive interactions with 30 species found from the ordinal regression to be highly abundant in responders (Fig. S2). Lastly, by integrating the species co-abundance network with the amino acid KO profile, we identified 6 significantly enriched species in responders (*C. symbiosum*,* B. cellulosilyticus*,* G. formicilis*,* F. saccharivorans*,* E. ramulus*,* D. longicatena*, and *E. hallii*) contributing to 22 amino acid-related KOs, which were further positively correlated with 6 AAA species (*R. gnavus*,* B. wadsworthia*,* B. adolescentis*,* D. formicigenerans*,* C. aerofaciens*, and *E. eligens*) (Fig. 3e).

In summary, our analysis revealed signature species in responders and non-responders that could serve as biomarkers of microbiome’s resistance to lifestyle interventions. Furthermore, the functional capacity of enriched species in responders suggest that amino acid biosynthesis is playing an important role in regulating microbiome dynamics.

#### Development of a machine learning model to predict microbiome responsiveness

We then explored whether a machine learning (ML) model can be developed for predicting the degree of responsiveness of a microbiome community to lifestyle interventions. We used the abundance of bacterial species, genera, and pathways from the baseline samples among the cohorts with lifestyle intervention as features for training the model. We used the baseline samples of subjects classified above as responders (*N* = 78) and non-responders (*N* = 47, Table S8). We built a total of four different gradient boosting machine (gbm) models depending on the input data to classify patients as responders or non-responders: a species, a genus, a taxonomic (with genus and species), and a hybrid model using pathways and taxa (Table S3).

**Fig. 4:**
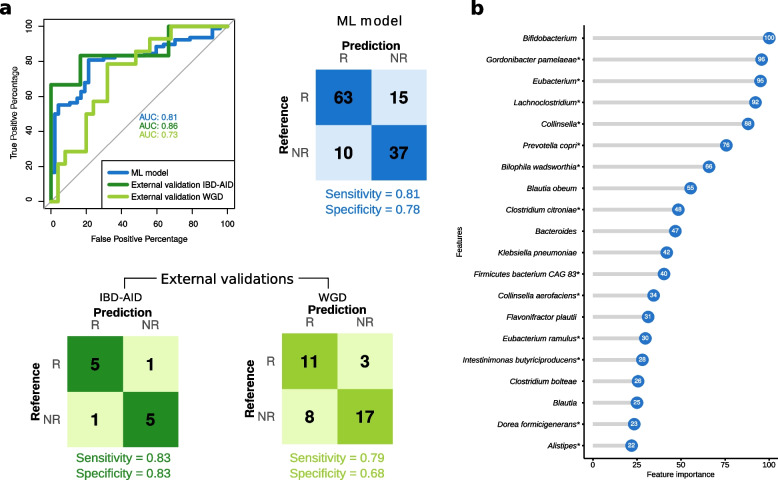
Performance of the machine learning model to classify individuals as responders and non-responders based on the degree of microbiome response. **a** Receiver operating characteristic curves (ROC) for the final model and external validation. Confusion matrix of the training model and external validation cohorts. IBD-AID: Inflammatory bowel disease-anti-inflammatory diet; WGD: whole grain diet. **b** Variance importance of the top 20 features selected by the final model. Significantly different in abundance species using ordinal regression are marked with *. The importance score of each feature is indicated inside the blue circles

We found that the species-based model classified the responders vs non-responders correctly, with an AUC of 0.75 ± 0.10. Eight species were selected in more than 70% of the 100 gbm species-based models, and *P. copri* (a significantly enriched species in non-responders) was selected in all the models. The classification performance was slightly increased in the genus-based model with an AUC of 0.79 ± 0.09. In the case of the genus-based model, 16 genera were consistently selected (> 70% of the 100 gbm models), and 2 were selected in all the models (*Bacteroides* and *Prevotella*). Similar classification performance was obtained when combining species and genus together (0.78 ± 0.08 AUC) or combining species with pathways (AUC of 0.74 ± 0.10). We built a final taxonomic-based model (see “Methods” for details) and obtained an AUC of 0.81 for the training set (sensitivity = 0.81 and specificity = 0.78, Fig. 4a). Recursive feature elimination was performed to reduce the dimensionality of the dataset to select the most important taxa for the classification of the responsiveness of the microbiome community. Figure 4b shows the feature importance of each of the selected features, or in other words, a score that measures how powerful is each feature in classifying the microbiome responsiveness. The final model consisted of 18 species and 12 genera as the top features, including 13 species and 6 genera that were significantly associated with responsiveness in the ordinal regression analysis (Fig. 4B, Table S4). The final model was then validated in two different external cohorts. The first cohort of subjects with inflammatory bowel disease (IBD) underwent an IBD-anti-inflammatory diet (IBD-AID, consumption of prebiotics, probiotics, and beneficial foods) for a period of 8 weeks, and the second cohort underwent a whole grain-rich diet (WGD) intervention for 8 weeks and consisted of overweight subjects. A total of 6 responders and 6 non-responders were identified in the IBD cohort, and 14 responders and 25 non-responders in the overweight cohort based on the same criteria established. The predictive power of the model in the external cohorts remained high with an AUC of 0.86 (sensitivity = 0.83 and specificity = 0.83) for the IBD-AID intervention and an AUC of 0.73 (sensitivity = 0.79 and specificity = 0.68) for the WGD intervention (Fig. 4A).

In summary, a gradient boosting model based on taxonomic data was developed achieving a good prediction of the microbiome response in two external cohorts including individuals from different ethnic backgrounds with metabolic and non-metabolic diseases that underwent lifestyle interventions.

## Discussion

From metabolic to immune to neurological disorders, the microbiome influences the development, progression, and therapeutic outcomes of diseases [27–30]. A novel treatment approach for both disease control and disease prevention involves altering host–microbiota interactions through tailored lifestyle interventions. Unlike antibiotics usage, which is broadly reported to have a negative impact on healthy host by significantly decreasing the overall gut microbiome diversity, lifestyle interventions are regarded as a beneficial strategy to improve the metabolic performance by modulating the host-gut microbiome. Changes in the composition of the bacterial consortia in the gut from a disease-associated to a more homeostatic state are one of the desired effects of lifestyle interventions. Furthermore, comparing with the dramatical dysregulation of gut microbiome after receiving high doses of antibiotics [15], although the response to lifestyle interventions may have a common signature within the population, heterogeneous and highly personalized shifts in the human microbiota have been confirmed in several studies [31–35].

Here we attempted to identify robust and generalizable biomarkers among the gut microbial communities associated with the degree of change in the microbiome structure. We performed longitudinal shotgun metagenomics analysis from a wide range of lifestyle interventions, and established criteria to classify individuals as responders and non-responders based on their gut microbiome restructuring, using as a point of departure the natural fluctuation of a healthy gut microbiome without any intervention. We identified *P. copri*, *B. stercoris*, and *B. vulgatus* to be highly abundant in the baseline microbiomes of individuals in whom lifestyle interventions had only a minor impact on the microbial community’s structure. Similarly, we found these 3 species enriched in the microbiome of individuals that were resistant to antibiotics treatment in line with recent evidence [36] from a 16S rRNA-based analysis in which the response to antibiotics in humans is determined by specific genera in the pre-treatment microbiota. Interestingly,* P. copri*, *B. stercoris*, and *B. vulgatus* are highly stable in the absence of interventions (ICCs > 0.5) suggesting their potential as biomarkers for microbiome stratification.

In contrast to the low number of species found enriched in the resistant microbiomes, we found 38 species to be highly abundant in the microbiomes that were significantly re-structured in response to lifestyle interventions. Interestingly, almost all species enriched in responders were positively correlated with at least one amino acid biosynthesis pathway. The interchange of vital metabolites, also known as metabolic cross-feeding, is a crucial process that controls the development and composition of microbial communities. Case-by-case explanations of the significance of amino acids in natural interkingdom and interspecies exchange networks have been provided by entomological investigations [37, 38]. Furthermore, a considerable proportion of all bacteria, according to comparative analysis of microbial genomes, lack crucial pathways for amino acid production [39]. Therefore, amino acid auxotrophy may promote cooperative interactions between different bacteria in the microbiome [25]. Our findings here suggest that microbiomes with a high abundance of amino acid biosynthesis pathways are also more likely to respond to different lifestyle interventions including both dietary and exercise interventions, targeting the restructuring of gut microbial communities. This finding is consistent with previous studies that amino acid biosynthesis is enriched in elite athletes [40] and decreased with high-fat diet treatment [41], highlighting the inner correlation between exercise and diet interventions. Therefore, supplementation with *F. prausnitzii*,* F. saccharivorans*,* E. ramulus*, and *E. hallii*, or other species both enriched and identified here as major taxonomic drivers of amino acid biosynthesis in the responder group, should be explored as a way to restore the metabolic flexibility required prior to microbiome-targeted lifestyle interventions. Importantly, among these potentially beneficial species, *F. prausnitzii*,* F. saccharivorans*, and *E. hallii* were reported to be enriched after exercise and diet intervention across multiple studies [42–47].

Similar to personalized medicine, personalized lifestyle approaches look for critical microbiome characteristics that can predict how an individual will react to specific lifestyle components. This information can then be used to help design a lifestyle that will have positive effects. Identifying the interactions between the host, the microbiome, and lifestyle exposures that influence lifestyle responses is the fundamental difficulty in realizing the potential of a microbiome-informed customized lifestyle. Whereas previous studies have demonstrated that the microbiome composition can be used to classify individuals into responders and non-responders on the basis of the health improvements from lifestyle interventions [48, 49], predictive models of personalized microbiota response have not yet been developed. We demonstrated here that it is possible to develop a generic ML model covering diverse lifestyle exposures that predicts the scale of microbiome change using only the baseline microbiome composition. Our model, which achieved AUCs up to 0.86 in external validation cohorts, can potentially be used for individual microbiome-based stratification, as an intermediate step towards personalized recommendations for improving the success rates of certain lifestyle interventions.

Our study has limitations. Even though several comparative analyses among studies have been performed using the ICC values [22], it is possible that ICCs may be affected by the general setup of each study, including the storage and sampling procedures, which may influence the outcome of the comparative analysis. Nevertheless, previous studies suggested a relatively stable bacterial community evaluated by ICCs with limited impact by the processing speed and storage duration [50, 51]. Furthermore, DNA extraction methods have been shown to influence the microbiome community results [52], and remained inconsistent across different cohorts in our study. Nevertheless, the impact of DNA extraction methods on metagenomic shotgun sequencing analysis of stool samples was reported to be the lowest compared to other tissue [53]. Lastly, following a strict filtering criterion, only two large-scale studies, both with Caucasian subjects, were selected to represent the healthy gut microbiome with high confidence of disease absence. Analysis of a larger cohort, well-balanced in gender and ethnicity, would allow to establish a more generalized baseline of microbiome variation in healthy individuals. The number of studies with dense longitudinal characterization of the microbiome upon lifestyle interventions is also limited and in most cases the clinical and biochemical data of the subjects are not available. Larger, more complete, and balanced datasets would allow to increase the statistical power of the data analysis and use of advanced algorithms, like deep learning, to investigate the correlation between microbiome and host response to lifestyle interventions. Nevertheless, our study offered novel insight into the microbial species and functions that may determine microbiome dynamics in response to lifestyle interventions.

## Conclusions

Human gut microbiome serves as a therapeutic target for multiple diseases through lifestyle interventions. However, subjects may have different treatment efficacy which may be due to the response of gut microbiota towards the interventions. In this study, we observe individuals with either highly plastic or resistant microbial composition with the stress of lifestyle interventions. We further identify key species and functions such as *Bacteroides stercoris*, *Prevotella copri*, and amino acid biosynthesis regulating the responsiveness of the gut microbiota. Last but not least, we demonstrate with our machine learning model that it is possible to predict microbiome resistance to change in response to lifestyle interventions using the baseline microbiome composition. In summary, this study shows that the composition and function of the gut microbiome are important to determine their response to lifestyle interventions and this knowledge may help to improve the design of personalized lifestyle approaches.

## Methods

### Data collection and availability

In this study, we collected shotgun metagenomic sequencing data from 10 publicly open available microbiome projects. These projects included (i) 2 longitudinal cohorts of healthy subjects (*N* = 231); (ii) 4 antibiotic intervention cohorts (*N* = 71); and (iii) 5 lifestyle intervention cohorts (*N* = 165) with metabolically diseased subjects that underwent dietary and/or exercise interventions (Table 1 and Table S5). The 2 longitudinal studies of healthy subjects with no intervention applied, abbreviated as CTL_1 and CTL_2, respectively, served as controls of normal gut microbiota fluctuation. In both studies, the selected subjects were not asked to follow diet or lifestyle recommendations and they followed their own lifestyle habits. From CTL_1, two pairs of samples taken 6 months apart from 140 subjects were used. In CTL_2, we used data from 78 subjects with one pair of samples and 4 subjects with a dense long-term time series. We used pair samples with a time interval between pairs of 2–3 months. We also selected samples that were taken 4 days apart (12 such pair samples were included). For the antibiotic intervention cohorts, the study of Palleja et al. [15] provides a cohort of healthy subjects that underwent a 4-day intervention with a cocktail of 3 last-resort antibiotics: meropenem, gentamicin, and vancomycin (A_MER–GEN–VAN). The Raymond et al. [54] cohort is composed of healthy participants that were treated twice a day with an oral dose of cefprozil for 7 days (A_CEF). The Willmann et al. [17] study provides two different cohorts of hematological patients receiving prophylactic antibiotics during a mean period of 6 days. One cohort was treated with ciprofloxacin (A_CIP) and the other with cotrimoxazole (A_COT). Regarding the lifestyle intervention cohorts, the first cohort was obtained from the study of Louis et al. [18] in which obese patients were involved in a multidisciplinary weight-loss program for 3 months (I_MWP). In Mardinoglu et al. [19], Non-alcoholic fatty liver disease (NAFLD) obese subjects underwent a low-carbohydrate diet with increased protein content during a 2-week period (I_LCD). The cohort of Zhao et al. [20] is composed of participants diagnosed with type 2 diabetes (T2D) that were administered a high-fiber diet for 3 months (I_HFD). The Ni et al. study provides data from NAFLD patients that were involved in a diet with high-resistant starch type II content for 4 months (I_HRS). The last cohort, from Liu et al. [9], is composed of prediabetes patients that enrolled in an exercise training program 3 days/week for a period of 3 months (I_ETP). More information and the number of samples used in each cohort are shown in Table 1 and Table S5. The Olendzki et al. [11] cohort was used as external validation of the machine learning predictive final model of response to lifestyle interventions. It is an IBD-anti-inflammatory dietary intervention (IBD-AID) for 8 weeks in a total of 15 subjects with inflammatory bowel disease. A second external validation cohort from Nielsen et al. [55] composed of 50 overweight subjects that underwent a whole grain dietary intervention for 8 weeks was used.

### Quality control and taxonomic profiling

For the quality control of the raw reads, human DNA contaminations were removed using bwa mem against the human reference genome ucsc.hg19, and adaptors, low-quality reads, bases, or PCR duplicates were filtered as previously described [56]. The high-quality reads were taxonomically profiled at different taxonomic levels using MetaPhlAn 3.0 [57]. Default settings were used to generate taxonomic relative abundances (total sum scaling normalization).

### Functional profiling

Microbial gene family abundances in metagenomic DNA reads were estimated using HUMAnN 3.0 [58]. Gene families were further mapped to the MetaCyc metabolic pathway database included in HUMAnN3 to obtain the MetaCyc pathway abundances. KOs with the species contribution were obtained in HUMAnN3 by KEGG database. Tables of pathway and gene family abundance obtained using HUMAnN3 were normalized to copies per million (CPM), including unmapped and unintegrated read mass.

### Microbiome diversity measurements

Microbiome diversity was calculated based on the species, phylum, and KO gene abundance profiles, respectively. For taxonomic diversity, 3 alpha diversity indexes (including Shannon diversity, Simpson diversity, Chao1 diversity) and 3 beta diversity indexes (including Bray–Curtis dissimilarity, Weighted and Unweighted UniFrac distance) were analyzed by the vegan package [59] and phyloseq package [60] in R, respectively. For functional diversity, the 3 mentioned alpha diversity indexes and Bray–Curtis dissimilarity were calculated. Principal coordinate analysis (PCoA) based on the beta diversity was performed, and the top 5 axes were included for follow-up analyses.

### ICCs of microbiome measurements

The intraclass correlation coefficient (ICC) that ranges from 0 to1 was used to represent the microbial stability (and resistance to perturbations) from totally unstable (ICC = 0) to perfectly stable (ICC = 1). We evaluated the ICC value for each diversity measurement described above and for the species, genus and MetaCyc pathway profiles using relative abundances to investigate the microbial stability within individuals of the no-intervention cohort and the resistance to perturbations within individuals from intervention cohorts. Diversity indexes were transformed into Gaussian distribution with bestNormalize package in R and the arcsine square-root transformation was implemented to the relative abundances of taxonomic and functional profiles as proposed previously [61]. After the metric transformation, ICC estimates and their 95% confident intervals were calculated using the rptR [62] package in R based on a mean-rating, absolute-agreement, 2-way random-effects model with 1000 bootstraps. The statistical comparisons of ICC values among cohort types were performed with Student *t* test. False discovery rate (FDR) correction was implemented to adjust *p* value for multiple comparisons.

### Defining degree of response to perturbation

We first calculated the Bray–Curtis dissimilarity of the microbiome composition between samples within 1–2 days for each individual from the longitudinal cohorts with no intervention, which we used to estimate the daily fluctuation of the microbiome without disturbance. We then evaluated the degree of response towards lifestyle (and antibiotic) interventions of each subject by calculating the Bray–Curtis dissimilarity between baseline and each time point after the intervention and selected the time point with the first peak value of Bray–Curtis distance to baseline. The information of the selected time point for each subject among studies are shown in Table S7. The mean + SD and the mean + 2SD of the Bray–Curtis dissimilarity calculated from the control cohorts (no-intervention) were further used as the two cut-offs in the lifestyle interventions for distinguishing between responders (> mean + 2SD), partial-responders ([mean + SD, mean + 2SD]) and non-responders (< mean + SD). PERMANOVA tests were performed among responders, partial-responders, and non-responders of the lifestyle interventions using the Bray–Curtis dissimilarity of baseline microbiome, and an ordinal regression model was used to find statistically significant taxonomic and functional differences among the three groups by applying the ordinal package in R. FDR correction was implemented to adjust *p* value for multiple comparisons.

### Network analysis

In order to investigate the differences and the role of specific taxa in the microbiome community between the subjects with different responses, network analyses were performed using taxonomic data of the baseline samples. To build SparCC correlation networks for the responder and non-responder subjects, FastSpar R package was used. Only significant correlations between species were considered (adjusted *p* < 0.1). Cytoscape version 3.9.0 [63] was used to analyze the networks. Statistical comparisons between the degree and closeness centrality of the taxonomic networks of responders and non-responders were performed using the t.test function from R package stats. Furthermore, the Spearman correlation among species significantly enriched in responders, their contributed KOs which were related to the biosynthesis of amino acid and AA auxotroph species were performed in R. Only significant correlations were considered (adjusted *p* < 0.1).

### Development of machine learning models

The Caret [64] R package was used to build a gradient boosting machine (gbm) model to train and classify responders and non-responders based on the baseline microbiome. We built 4 different models depending on the input data provided: a species model, a genus model, a taxonomic model using species and genus data, and a hybrid model using species, genus, and pathways data. To obtain a learning model with good interpretability and generalizability, we built a final model that included not only internal validation but also external validations, as it is critical to developing quality machine learning models [65]. The following approach was applied to build the model which included the following steps: (1) loaded the specific data (depending on the model species, genera, or pathways); (2) used the createdatapartition function from caret package to select 80% of the samples as training set; (3) performed feature selection in the training set selecting the top 30 features by applying recursive feature elimination using the rfe R function; (4) trained the model after centering, scaling the data, and removing variables with near-zero variance, using leave-one-out cross-validation (LOOCV) as a resampling method. Leave-one-out cross-validation (LOOCV) is a special case of *K*-fold cross-validation, where *K* equals the number of observations in the dataset [66]. Cross-validation techniques are used for evaluating ML models protecting the model against overfitting or selection bias and giving insights on how the model will generalize when an independent dataset is provided to the model. GBM was used as a machine learning model method and grid search to tune the hyperparameters. “Interaction.depth”, “n.trees”, “shrinkage”, and “n.minobsinnode” were applied by the expand.grid R function; (5) tested the training model in the 20% of the data. Doing only one partition may provide biased results depending on the data split (“lucky” or “unlucky” split) [67]. Therefore, in order to perform a robust interpretation of the model’s performance, the machine learning algorithm was applied 100 times using different random training-test splits; (6) steps 2–5 were repeated 99 times to obtain the overall testing performances. Model performance was assessed using the evalm function from Mleval R package, and receiver operating characteristic curve (ROC) was obtained using the R package pROC; (7) then applied steps 3–4 to the entire dataset to obtain the final machine learning model; (8) evaluated the model’s performance in external cohorts (information about the external cohorts is found in the “Supplementary Information” section).

### Data visualization

The circos plot was made using iTOL (interactive Tree of Life) v6 [68]. Network visualizations were made by using the software Cytoscape version 3.9.0 [63]. All the other figures were generated by R software 3.6.3, using ggplot2, ggcorrplot, and pROC packages.

### Supplementary Information

**Additional file 1: Figure S1.** Related to Figure 3. Relative abundances of the significant pathways using ordinal regression among non-responders, partly-responders and responders groups (*p* < 0.05). **Figure S2.** Related to Figure 3. Correlation network of responders showing the positive correlations between enriched in responders species and auxotroph species (only significant correlations are considered, *p* < 0.05). Width and color intensity or the edges refers to the correlation value. Blue nodes are species significantly enriched in responders, yellow nodes are AA auxotroph species and orange nodes are AA auxotroph and significantly enriched in responders species. **Table S1.** Related to Figure 1. Detailed ICCs value of different diversity indexes for each cohort. **Table S2.** Related to Figure 4. Statistics of the ICCs value of each species. Uniquely influenced disease related species of each cohort. **Table S3.** Related to Figure 4. Model performance results of the 100 different splits. Mean and standard deviation of sensitivity, specificity, and AUC for the 100 models. **Table S4.** Related to Figure 4. Species and genus selected by the final model. Significance from the ordinal regression comparing response groups. No: non-significant, Enriched R: significant and enriched in responders, Enriched NR: significant and enriched in non-responders. **Table S5.** Related to Table 1. Summary of sequencing and microbiome information of the studies used in the meta-analysis. **Table S6.** Related to Figure 2. Significant species between responder and non-responder from ordinal regression. **Table S7.** Related to Table 1. Information of the time point selected for each subject for responsiveness classification. **Table S8.** Related to Figure 4. Count of each category among discovery and validation cohorts.

## Data Availability

The raw sequence data used in the current study are available in the NCBI Sequencing Read Archive under the accession numbers: PRJNA354235, PRJNA544527, PRJEB20800, PRJEB8094, PRJEB28058, PRJNA290729, PRJNA420817, PRJEB15179, PRJNA703757, PRJNA454826, PRJNA642308, and PRJNA395744.
