# Supplementary material for: Identification of robust and generalizable biomarkers for microbiome-based stratification in lifestyle interventions
Source: Microbiome. 2023 Aug 8;11:178. doi: 10.1186/s40168-023-01604-z (PMC10408196; doi:10.1186/s40168-023-01604-z)
Supplement: Supplementary file 2 — Additional file 1: Figure S1. Related to Figure 3. Relative abundances of the significant pathways using ordinal regression among non-responders, partly-responders and responders groups (p < 0.05). Figure S2. Related to Figure 3. Correlation network of responders showing the positive correlations between enriched in responders species and auxotroph species (only significant correlations are considered, p < 0.05). Width and color intensity or the edges refers to the correlation value. Blue nodes are species significantly enriched in responders, yellow nodes are AA auxotroph species and orange nodes are AA auxotroph and significantly enriched in responders species. Table S1. Related to Figure 1. Detailed ICCs value of different diversity indexes for each cohort. Table S2. Related to Figure 4. Statistics of the ICCs value of each species. Uniquely influenced disease related species of each cohort. Table S3. Related to Figure 4. Model performance results of the 100 different splits. Mean and standard deviation of sensitivity, specificity, and AUC for the 100 models. Table S4. Related to Figure 4. Species and genus selected by the final model. Significance from the ordinal regression comparing response groups. No: non-significant, Enriched R: significant and enriched in responders, Enriched NR: significant and enriched in non-responders. Table S5. Related to Table 1. Summary of sequencing and microbiome information of the studies used in the meta-analysis. Table S6. Related to Figure 2. Significant species between responder and non-responder from ordinal regression. Table S7. Related to Table 1. Information of the time point selected for each subject for responsiveness classification. Table S8. Related to Figure 4. Count of each category among discovery and validation cohorts. [file 40168_2023_1604_MOESM1_ESM.docx]

**SUPPLEMENTAL INFORMATION**


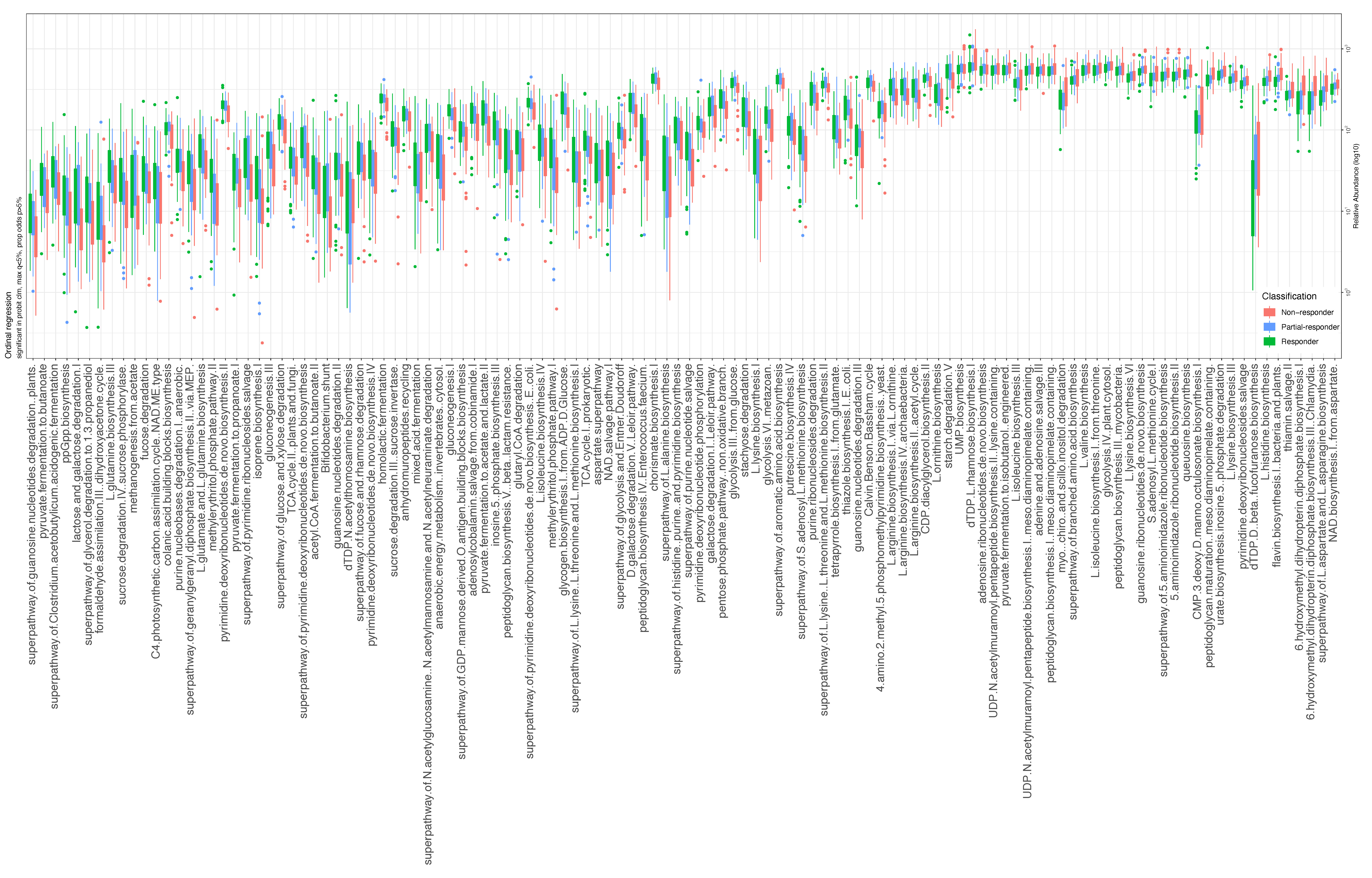


**Figure S1. Related to Figure 3.** Relative abundances of the significant pathways using ordinal regression among non-responders, partly-responders and responders groups (p < 0.05).





**Figure S2. Related to Figure 3.** Correlation network of responders showing the positive correlations between enriched in responders species and auxotroph species (only significant correlations are considered, p < 0.05). Width and color intensity or the edges refers to the correlation value. Blue nodes are species significantly enriched in responders, yellow nodes are AA auxotroph species and orange nodes are AA auxotroph and significantly enriched in responders species.

**Table S1. Related to Figure 1.** Detailed ICCs value of different diversity indexes for each cohort.

| **ICC Value of Diversity Index** | **CTL_1** | **CTL_2** | **A_MER-GEN-VAN** | **A_CEF** | **A_CIP** | **A_COT** | **I_MWP** | **I_LCD** | **I_HFD** | **I_HRS** | **I_ETP** |
| --- | --- | --- | --- | --- | --- | --- | --- | --- | --- | --- | --- |
| **Observed OTU** | 0.41 | 0.58 | 0.00 | 0.18 | 0.28 | 0.00 | 0.00 | 0.85 | 0.58 | 0.44 | 0.43 |
| **Shannon Diversity** | 0.70 | 0.70 | 0.00 | 0.46 | 0.42 | 0.00 | 0.31 | 0.69 | 0.35 | 0.45 | 0.44 |
| **Simpson Diversity** | 0.71 | 0.66 | 0.00 | 0.39 | 0.35 | 0.00 | 0.21 | 0.61 | 0.25 | 0.40 | 0.45 |
| **Bray-Curtis PCoA1** | 0.82 | 0.59 | 0.00 | 0.69 | 0.52 | 0.01 | 0.29 | 0.46 | 0.61 | 0.70 | 0.72 |
| **Average of Bray-Curtis PCoA1-5** | 0.77 | 0.58 | 0.20 | 0.61 | 0.28 | 0.25 | 0.19 | 0.18 | 0.38 | 0.38 | 0.61 |
| **Unweighted Unifrac PCoA1** | 0.83 | 0.55 | 0.00 | 0.76 | 0.52 | 0.00 | 0.48 | 0.65 | 0.77 | 0.39 | 0.51 |
| **Average of Unweighted Unifrac PCoA1-5** | 0.71 | 0.66 | 0.32 | 0.49 | 0.51 | 0.41 | 0.36 | 0.57 | 0.49 | 0.42 | 0.31 |
| **Weighted Unifrac PCoA1** | 0.66 | 0.65 | 0.16 | 0.62 | 0.37 | 0.22 | 0.31 | 0.33 | 0.35 | 0.45 | 0.64 |
| **Average of Weighted Unifrac PCoA1-5** | 0.73 | 0.41 | 0.14 | 0.32 | 0.32 | 0.06 | 0.16 | 0.27 | 0.29 | 0.40 | 0.31 |

**Table S2. Related to Figure 4.** Statistics of the ICCs value of each species. Uniquely influenced disease related species of each cohort

| **Cohort** | **Species** |
| --- | --- |
| **I_MWP** | *Coprobacter fastidiosus* |
|  | *Christensenella minuta* |
|  | *Prevotella copri* |
|  | *Gemmiger formicilis* |
|  | *Alistipes shahii* |
|  | *Bacteroides coprocola* |
| **I_LCD** | *Bifidobacterium catenulatum* |
|  | *Bifidobacterium animalis* |
|  | *Lactobacillus rhamnosus* |
|  | *Prevotella corporis* |
|  | *Peptostreptococcus anaerobius* |
|  | *Clostridium saccharolyticum* |
|  | *Bacteroides plebeius* |
|  | *Prevotella disiens* |
| **I_HFD** | *Clostridium perfringens* |
|  | *Enterococcus faecium* |
|  | *Prevotella buccae* |
|  | *Lactobacillus crispatus* |
|  | *Pyramidobacter piscolens* |
|  | *Bacteroides clarus* |
|  | *Streptococcus oralis* |
|  | *Lactobacillus salivarius* |
|  | *Parabacteroides johnsonii* |
|  | *Lactobacillus mucosae* |
|  | *Blautia coccoides* |
|  | *Bifidobacterium bifidum* |
| **I_HRS** | *Ruminococcus callidus* |
|  | *Desulfovibrio piger* |
|  | *Clostridium clostridioforme* |
| **I_ETP** | *Prevotella bivia* |

**Table S3. Related to Figure 4.** Model performance results of the 100 different splits. Mean and standard deviation of sensitivity, specificity, and AUC for the 100 models

| **Model** | **Sensitivity** | **Specificity** | **AUC** |
| --- | --- | --- | --- |
| **Species** | 0.74 ± 0.20 | 0.78± 0.18 | 0.75 ± 0.10 |
| **Genus** | 0.75 ± 0.16 | 0.84 ± 0.12 | 0.79 ± 0.09 |
| **Taxonomic** | 0.78 ± 0.13 | 0.79 ± 0.13 | 0.78 ± 0.08 |
| **Hybrid** | 0.73 ± 0.18 | 0.79 ± 0.16 | 0.74 ± 0.10 |

**Table S4. Related to Figure 4.** Species and genus selected by the final model. Significance from the ordinal regression comparing response groups. No: non-significant, Enriched R: significant and enriched in responders, Enriched NR: significant and enriched in non-responders.

| **Features** | **Significant in ordinal regression** |
| --- | --- |
| *Lachnoclostridium* | Enriched R |
| *Prevotella copri* | Enriched NR |
| *Bifidobacterium* | No |
| *Prevotella* | Enriched NR |
| *Collinsella* | Enriched R |
| *Acidaminococcus* | No |
| *Bacteroides* | No |
| *Gordonibacter pamelaeae* | Enriched R |
| *Eubacterium* | Enriched R |
| *Eubacterium ramulus* | Enriched R |
| *Bacteroides intestinalis* | Enriched R |
| *Bilophila wadsworthia* | Enriched R |
| *Eisenbergiella* | Enriched R |
| *Clostridium citroniae* | Enriched R |
| *Clostridium asparagiforme* | Enriched R |
| *Bacteroides cellulosilyticus* | Enriched R |
| *Bifidobacterium bifidum* | Enriched R |
| *Dorea formicigenerans* | Enriched R |
| *Blautia* | No |
| *Flavonifractor plautii* | No |
| *Firmicutes bacterium CAG 83* | Enriched R |
| *Klebsiella pneumoniae* | No |
| *Collinsella aerofaciens* | Enriched R |
| *Alistipes* | Enriched R |
| *Clostridium bolteae* | No |
| *Mitsuokella multacida* | No |
| *Intestinimonas butyriciproducens* | Enriched R |
| *Blautia obeum* | No |
| *Lactobacillus* | No |
| *Streptococcus infantis* | No |

**Table S5. Related to Table 1.** Summary of sequencing and microbiome information of the studies used in the meta-analysis.

| **Study** | | **Average Read Counts** | **Library Preparation** | **Sequencing Platform** | **Company** | **DNA extraction Kits** | **Bead-beating** | **MetaPhlAn3 (species)** | **KOs** | **MetaCyc** | **Age (Years)** | **Gender (M/F)** | **BMI (kg/m²)** | **Ethnicity** | **Time between samples (days)** |
| --- | --- | --- | --- | --- | --- | --- | --- | --- | --- | --- | --- | --- | --- | --- | --- |
|  | **HEALTHY CONTROLS** | | | | | | | | | | | | | |  |
| Mehta et al. | | 10842582 | Nextera XT  100 read length | Illumina | - | MO BIO PowerLyzer kit | Yes | 73 ± 14 | 1688 ± 397 | 265 ± 44 | [65-81] median 69 | 140/0 | - | Mostly Caucasian | 1-3 |
| Poyet et al. | | 9409512 | Nextera  100 read length | Illumina | Broad Institute | DNeasy UltraClean96 MicrobioalKit, PureLinkPro96_gDNAkit | Yes | 55 ± 12 | 1442 ± 342 | 245 ± 37 | 28 ± 6 | 59/23 | 23 ± 3 | Caucasian | 15 ± 26 |
|  | **ANTIBIOTIC INTERVENTIONS** | | | | | | | | | | | | | |  |
| Palleja et al. | | 19870883 | -  100 read length | Illumina | BGI-Shenzhen | - | Yes | 80 ± 25 | 2383 ± 553 | 326 ± 48 | 23 ± 4 | 9/0 | >18.5, <25 | Caucasian | 4/8 |
| Raymond et al. | | 19939997 | Nextera  100 read length | Illumina | - | MO BIO PowerMax Soil DNA | Yes | 66 ± 18 | 1607 ± 392 | 266 ± 44 | 26 ± 4 | 9/9 | 25 ± 3 | Caucasian | 3/5/7 |
| Willmann et al. | | 19639553 | NEBNext  150 read length | Illumina | GATC Biotech AG | MO BIO PowerSoil DNA | Yes | 56 ± 23 | 1599 ± 630 | 270 ± 62 | >18 | Both | - | Caucasian | 1/3/6 |
|  | **LIFESTYLE INTERVENTIONS** | | | | | | | | | | | | | |  |
| Louis et al. | | 10994668 | Nextera  100 read length | Illumina | CeGat | PSP Spin Stool DNA | Yes | 62 ± 11 | 1345 ± 266 | 238 ± 28 | 40 ± 8 | 7/9 | 43 ± 7 | Caucasian | 84 |
| Mardinoglu et al. | | 17432569 | -  150 read length | Illumina | Genomics Core Facility of Univ. of Gothenburg | QIAamp DNA mini stool kit | Yes | 85 ± 16 | 1936 ± 333 | 297 ± 34 | [20-70] | 8/2 | 34 ± 1 | Caucasian | 1/3/7/14 |
| Zhao et al. | | 19824782 | -  150 read length | Illumina | GENEWIZ Co. | QIAamp DNA mini stool kit | Yes | 88 ± 17 | 2256 ± 438 | 332 ± 38 | [37-70] | Both | - | Asian | 84 |
| Ni et al. | | 17029920 | 150 read length | Illumina | Novogene | PSP Spin Stool DNA | Yes | 60 ± 17 | 2183 ± 524 | 323 ± 42 | 39 ± 9 | 37/13 | 28 ± 3 | Asian | 120 |
| Liu et al. | | 13858615 | -  150 read length | Illumina | BGI | PSP Spin Stool DNA | Yes | 89 ± 12 | 2058 ± 469 | 315 ± 38 | 43 ± 11 | 20/0 | 29 ± 4 | Asian | 84 |
|  | **External validations** | | | | | | | | | | | | | |  |
| Olendzki et al. | | 3059370 | Nextera XT  150 read length | Illumina | - | MagAttract PowerSoil DNA | Yes | 31 ± 14 | 857 ± 509 | 138 ± 88 | 40.5 ± 12.8 | 7/8 | 39 ± 14 | Americans | 54 |
| Nielsen et al. | | 39716306 | -  150 read length | Illumina | - | - | Yes | 105 ± 16 | 2279 ± 408 | 298 ± 34 | [18-64] | Both | [25-35] | Caucasian | 54 |

**Table S6. Related to Figure 2.** Significant species between responder and non-responder from ordinal regression

| **species** | **estimate** | **std.error** | **statistic** | **p.value** | **q.value** |
| --- | --- | --- | --- | --- | --- |
| Prevotella_copri | -0.018319099 | 0.004567812 | -4.01047576 | 6.06E-05 | 0.009271268 |
| Gordonibacter_pamelaeae | 5.646198737 | 1.74476667 | 3.236076683 | 0.001211848 | 0.075363602 |
| Eubacterium_ramulus | 2.316868453 | 0.748110007 | 3.096962253 | 0.001955148 | 0.075363602 |
| Fusicatenibacter_saccharivorans | 0.237914075 | 0.076878547 | 3.094674457 | 0.00197029 | 0.075363602 |
| Collinsella_aerofaciens | 0.216556248 | 0.076163619 | 2.843302996 | 0.00446486 | 0.112518198 |
| Eubacterium_hallii | 0.533510983 | 0.188787961 | 2.825979898 | 0.004713622 | 0.112518198 |
| Bilophila_wadsworthia | 2.320701335 | 0.829613074 | 2.797329751 | 0.005152691 | 0.112518198 |
| Dorea_longicatena | 0.522563261 | 0.190762022 | 2.739346411 | 0.006156147 | 0.112518198 |
| Dorea_formicigenerans | 1.068727037 | 0.398333995 | 2.682992292 | 0.007296667 | 0.112518198 |
| Intestinimonas_butyriciproducens | 6.802825571 | 2.571219366 | 2.64575853 | 0.008150798 | 0.112518198 |
| Ruthenibacterium_lactatiformans | 0.622804137 | 0.237578033 | 2.621471896 | 0.008755098 | 0.112518198 |
| Odoribacter_splanchnicus | 0.45649763 | 0.174318124 | 2.618761718 | 0.008824957 | 0.112518198 |
| Alistipes_putredinis | 0.043295218 | 0.017183096 | 2.519640205 | 0.011747484 | 0.129946428 |
| Eubacterium_sp_CAG_274 | 0.589396701 | 0.236177339 | 2.495568386 | 0.012575551 | 0.129946428 |
| Oscillibacter_sp_CAG_241 | 2.134379089 | 0.856850003 | 2.490960007 | 0.012739846 | 0.129946428 |
| Bacteroides_intestinalis | 0.36073133 | 0.149202688 | 2.417726752 | 0.0156178 | 0.149345211 |
| Clostridium_sp_CAG_58 | 0.93765239 | 0.400791195 | 2.339503466 | 0.019309392 | 0.165564843 |
| Agathobaculum_butyriciproducens | 0.822315778 | 0.354793772 | 2.317728897 | 0.02046406 | 0.165564843 |
| Roseburia_faecis | 0.068159036 | 0.029749782 | 2.291076782 | 0.021958973 | 0.165564843 |
| Erysipelatoclostridium_ramosum | 1.688331918 | 0.738169231 | 2.28718815 | 0.022184843 | 0.165564843 |
| Turicibacter_sanguinis | 21.86605473 | 9.706499796 | 2.252722937 | 0.024276624 | 0.165564843 |
| Alistipes_shahii | 0.626788277 | 0.28055982 | 2.234062875 | 0.025478936 | 0.165564843 |
| Clostridium_citroniae | 3.200936475 | 1.457612066 | 2.19601398 | 0.028090943 | 0.165564843 |
| Eubacterium_sp_CAG_251 | 0.616979499 | 0.28181791 | 2.189284205 | 0.02857619 | 0.165564843 |
| Firmicutes_bacterium_CAG_83 | 0.362883069 | 0.169259238 | 2.143948379 | 0.032037023 | 0.165564843 |
| Oscillibacter_sp_57_20 | 0.2241205 | 0.104876809 | 2.136988161 | 0.032598955 | 0.165564843 |
| Clostridium_symbiosum | 3.666541175 | 1.730410104 | 2.118885671 | 0.03410013 | 0.165564843 |
| Clostridium_asparagiforme | 6.580139817 | 3.118610424 | 2.109958899 | 0.034861896 | 0.165564843 |
| Adlercreutzia_equolifaciens | 3.276986835 | 1.557708971 | 2.103722131 | 0.035402693 | 0.165564843 |
| Gemmiger_formicilis | 1.358596679 | 0.651837679 | 2.08425613 | 0.037136876 | 0.165564843 |
| Bacteroides_cellulosilyticus | 0.687904505 | 0.330462053 | 2.081644471 | 0.037374956 | 0.165564843 |
| Roseburia_intestinalis | 0.158785705 | 0.076330739 | 2.080232767 | 0.037504188 | 0.165564843 |
| Faecalibacterium_prausnitzii | 0.058509275 | 0.028140322 | 2.079197032 | 0.037599244 | 0.165564843 |
| Bacteroides_vulgatus | -0.014580945 | 0.007018584 | -2.077476666 | 0.037757587 | 0.165564843 |
| Holdemanella_biformis | 0.308543063 | 0.148630347 | 2.075908914 | 0.037902376 | 0.165564843 |
| Bacteroides_stercoris | -0.020207083 | 0.009787187 | -2.064646779 | 0.038956434 | 0.165564843 |
| Eggerthella_lenta | 1.82650889 | 0.892180532 | 2.047241366 | 0.040634392 | 0.168028701 |
| Bifidobacterium_bifidum | 1.754372228 | 0.870479171 | 2.015409774 | 0.043861731 | 0.176601181 |
| Bifidobacterium_pseudocatenulatum | 0.111308555 | 0.056067698 | 1.985252785 | 0.047116354 | 0.184841082 |
| Asaccharobacter_celatus | 2.893941534 | 1.470075736 | 1.968566287 | 0.049002917 | 0.18499732 |
| Anaerotruncus_colihominis | 5.09738238 | 2.59591387 | 1.963617683 | 0.049574445 | 0.18499732 |

**Table S7. Related to Table 1.** Information of the time point selected for each subject for responsiveness classification

| **Study** | **Subject_ID** | **Baseline_Time_Point** | **After_Treatment_Time_Point** |
| --- | --- | --- | --- |
| A_MER-GEN-VAN | S10 | Day0 | Day8 |
| A_MER-GEN-VAN | S11 | Day0 | Day8 |
| A_MER-GEN-VAN | S12 | Day0 | Day4 |
| A_MER-GEN-VAN | S1 | Day0 | Day8 |
| A_MER-GEN-VAN | S2 | Day0 | Day4 |
| A_MER-GEN-VAN | S3 | Day0 | Day8 |
| A_MER-GEN-VAN | S4 | Day0 | Day8 |
| A_MER-GEN-VAN | S5 | Day0 | Day4 |
| A_MER-GEN-VAN | S6 | Day0 | Day4 |
| A_MER-GEN-VAN | S7 | Day0 | Day4 |
| A_MER-GEN-VAN | S8 | Day0 | Day8 |
| A_MER-GEN-VAN | S9 | Day0 | Day8 |
| A_CEF | P10 | Day0 | Day7 |
| A_CEF | P11 | Day0 | Day7 |
| A_CEF | P12 | Day0 | Day7 |
| A_CEF | P13 | Day0 | Day7 |
| A_CEF | P14 | Day0 | Day7 |
| A_CEF | P15 | Day0 | Day7 |
| A_CEF | P17 | Day0 | Day7 |
| A_CEF | P18 | Day0 | Day7 |
| A_CEF | P19 | Day0 | Day7 |
| A_CEF | P1 | Day0 | Day7 |
| A_CEF | P20 | Day0 | Day7 |
| A_CEF | P21 | Day0 | Day7 |
| A_CEF | P22 | Day0 | Day7 |
| A_CEF | P2 | Day0 | Day7 |
| A_CEF | P3 | Day0 | Day7 |
| A_CEF | P4 | Day0 | Day7 |
| A_CEF | P5 | Day0 | Day7 |
| A_CEF | P9 | Day0 | Day7 |
| A_COT | 1 | Day0 | Day3 |
| A_COT | 3 | Day0 | Day1 |
| A_COT | 4 | Day0 | Day1 |
| A_COT | 5 | Day0 | Day3 |
| A_COT | 7 | Day0 | Day1 |
| A_COT | 8 | Day0 | Day6 |
| A_COT | 10 | Day0 | Day6 |
| A_COT | 11 | Day0 | Day3 |
| A_COT | 12 | Day0 | Day3 |
| A_COT | 13 | Day0 | Day3 |
| A_COT | 17 | Day0 | Day1 |
| A_COT | 19 | Day0 | Day1 |
| A_COT | 20 | Day0 | Day3 |
| A_COT | 21 | Day0 | Day3 |
| A_COT | 23 | Day0 | Day3 |
| A_COT | 24 | Day0 | Day1 |
| A_COT | 25 | Day0 | Day1 |
| A_COT | 26 | Day0 | Day3 |
| A_COT | 27 | Day0 | Day6 |
| A_COT | 28 | Day0 | Day6 |
| A_COT | 29 | Day0 | Day3 |
| A_CIP | 504 | Day0 | Day6 |
| A_CIP | 505 | Day0 | Day6 |
| A_CIP | 506 | Day0 | Day6 |
| A_CIP | 507 | Day0 | Day3 |
| A_CIP | 508 | Day0 | Day3 |
| A_CIP | 510 | Day0 | Day6 |
| A_CIP | 511 | Day0 | Day3 |
| A_CIP | 512 | Day0 | Day6 |
| A_CIP | 516 | Day0 | Day3 |
| A_CIP | 517 | Day0 | Day3 |
| A_CIP | 518 | Day0 | Day6 |
| A_CIP | 519 | Day0 | Day6 |
| A_CIP | 522 | Day0 | Day6 |
| A_CIP | 523 | Day0 | Day3 |
| A_CIP | 524 | Day0 | Day3 |
| A_CIP | 528 | Day0 | Day3 |
| A_CIP | 532 | Day0 | Day1 |
| A_CIP | 534 | Day0 | Day3 |
| A_CIP | 537 | Day0 | Day3 |
| A_CIP | 538 | Day0 | Day3 |
| I_MWP | AS44 | Week1 | Week12 |
| I_MWP | AS45 | Week1 | Week12 |
| I_MWP | AS50 | Week1 | Week12 |
| I_MWP | AS51 | Week1 | Week12 |
| I_MWP | AS53 | Week1 | Week12 |
| I_MWP | AS56 | Week1 | Week12 |
| I_MWP | AS58 | Week1 | Week12 |
| I_MWP | AS60 | Week1 | Week12 |
| I_MWP | AS62 | Week1 | Week12 |
| I_MWP | AS63 | Week1 | Week12 |
| I_MWP | AS64 | Week1 | Week12 |
| I_MWP | AS65 | Week1 | Week12 |
| I_MWP | AS66 | Week1 | Week12 |
| I_MWP | AS68 | Week1 | Week12 |
| I_LCD | 30314 | Day0 | Day14 |
| I_LCD | 30305 | Day0 | Day14 |
| I_LCD | 30302 | Day0 | Day7 |
| I_LCD | 30303 | Day0 | Day14 |
| I_LCD | 30312 | Day0 | Day14 |
| I_LCD | 30301 | Day0 | Day7 |
| I_LCD | 30316 | Day0 | Day1 |
| I_LCD | 30313 | Day0 | Day7 |
| I_LCD | 30310 | Day0 | Day3 |
| I_LCD | 30309 | Day0 | Day14 |
| I_HFD | 2 | Week1 | Week12 |
| I_HFD | 3 | Week1 | Week12 |
| I_HFD | 4 | Week1 | Week12 |
| I_HFD | 5 | Week1 | Week12 |
| I_HFD | 6 | Week1 | Week12 |
| I_HFD | 7 | Week1 | Week12 |
| I_HFD | 8 | Week1 | Week12 |
| I_HFD | 9 | Week1 | Week12 |
| I_HFD | 10 | Week1 | Week12 |
| I_HFD | 11 | Week1 | Week12 |
| I_HFD | 12 | Week1 | Week12 |
| I_HFD | 14 | Week1 | Week12 |
| I_HFD | 15 | Week1 | Week12 |
| I_HFD | 17 | Week1 | Week12 |
| I_HFD | 18 | Week1 | Week12 |
| I_HFD | 19 | Week1 | Week12 |
| I_HFD | 20 | Week1 | Week12 |
| I_HFD | 21 | Week1 | Week12 |
| I_HFD | 22 | Week1 | Week12 |
| I_HFD | 23 | Week1 | Week12 |
| I_HFD | 24 | Week1 | Week12 |
| I_HFD | 25 | Week1 | Week12 |
| I_HFD | 26 | Week1 | Week12 |
| I_HFD | 27 | Week1 | Week12 |
| I_HFD | 28 | Week1 | Week12 |
| I_HFD | 29 | Week1 | Week12 |
| I_HFD | 30 | Week1 | Week12 |
| I_HFD | 31 | Week1 | Week12 |
| I_HFD | 32 | Week1 | Week12 |
| I_HFD | 33 | Week1 | Week12 |
| I_HFD | 34 | Week1 | Week12 |
| I_HFD | 36 | Week1 | Week12 |
| I_HFD | 37 | Week1 | Week12 |
| I_HFD | 38 | Week1 | Week12 |
| I_HFD | 39 | Week1 | Week12 |
| I_HFD | 40 | Week1 | Week12 |
| I_HFD | 42 | Week1 | Week12 |
| I_HFD | 43 | Week1 | Week12 |
| I_HFD | 46 | Week1 | Week12 |
| I_HFD | 47 | Week1 | Week12 |
| I_HFD | 48 | Week1 | Week12 |
| I_HFD | 49 | Week1 | Week12 |
| I_HFD | 50 | Week1 | Week12 |
| I_HFD | 51 | Week1 | Week12 |
| I_HFD | 52 | Week1 | Week12 |
| I_HFD | 53 | Week1 | Week12 |
| I_HFD | 54 | Week1 | Week12 |
| I_HFD | 55 | Week1 | Week12 |
| I_HFD | 57 | Week1 | Week12 |
| I_HFD | 58 | Week1 | Week12 |
| I_HFD | 59 | Week1 | Week12 |
| I_HFD | 60 | Week1 | Week12 |
| I_HFD | 61 | Week1 | Week12 |
| I_HFD | 62 | Week1 | Week12 |
| I_HFD | 63 | Week1 | Week12 |
| I_HFD | 64 | Week1 | Week12 |
| I_HFD | 65 | Week1 | Week12 |
| I_HFD | 67 | Week1 | Week12 |
| I_HFD | 68 | Week1 | Week12 |
| I_HFD | 70 | Week1 | Week12 |
| I_HFD | 71 | Week1 | Week12 |
| I_HFD | 72 | Week1 | Week12 |
| I_HFD | 73 | Week1 | Week12 |
| I_HFD | 74 | Week1 | Week12 |
| I_HFD | 75 | Week1 | Week12 |
| I_HFD | 77 | Week1 | Week12 |
| I_HFD | 78 | Week1 | Week12 |
| I_HFD | 79 | Week1 | Week12 |
| I_HFD | 80 | Week1 | Week12 |
| I_HFD | 81 | Week1 | Week12 |
| I_HFD | 83 | Week1 | Week12 |
| I_HRS | RS003 | D0 | Day120 |
| I_HRS | RS010 | D0 | Day120 |
| I_HRS | RS011 | D0 | Day120 |
| I_HRS | RS012 | D0 | Day120 |
| I_HRS | RS018 | D0 | Day120 |
| I_HRS | RS023 | D0 | Day120 |
| I_HRS | RS025 | D0 | Day120 |
| I_HRS | RS026 | D0 | Day120 |
| I_HRS | RS027 | D0 | Day120 |
| I_HRS | RS034 | D0 | Day120 |
| I_HRS | RS041 | D0 | Day120 |
| I_HRS | RS046 | D0 | Day120 |
| I_HRS | RS053 | D0 | Day120 |
| I_HRS | RS054 | D0 | Day120 |
| I_HRS | RS058 | D0 | Day120 |
| I_HRS | RS061 | D0 | Day120 |
| I_HRS | RS063 | D0 | Day120 |
| I_HRS | RS064 | D0 | Day120 |
| I_HRS | RS069 | D0 | Day120 |
| I_HRS | RS073 | D0 | Day120 |
| I_HRS | RS075 | D0 | Day120 |
| I_HRS | RS076 | D0 | Day120 |
| I_HRS | RS080 | D0 | Day120 |
| I_HRS | RS081 | D0 | Day120 |
| I_HRS | RS089 | D0 | Day120 |
| I_HRS | RS100 | D0 | Day120 |
| I_HRS | RS101 | D0 | Day120 |
| I_HRS | RS109 | D0 | Day120 |
| I_HRS | RS114 | D0 | Day120 |
| I_HRS | RS116 | D0 | Day120 |
| I_HRS | RS119 | D0 | Day120 |
| I_HRS | RS122 | D0 | Day120 |
| I_HRS | RS124 | D0 | Day120 |
| I_HRS | RS125 | D0 | Day120 |
| I_HRS | RS129 | D0 | Day120 |
| I_HRS | RS138 | D0 | Day120 |
| I_HRS | RS143 | D0 | Day120 |
| I_HRS | RS149 | D0 | Day120 |
| I_HRS | RS150 | D0 | Day120 |
| I_HRS | RS151 | D0 | Day120 |
| I_HRS | RS158 | D0 | Day120 |
| I_HRS | RS162 | D0 | Day120 |
| I_HRS | RS167 | D0 | Day120 |
| I_HRS | RS173 | D0 | Day120 |
| I_HRS | RS174 | D0 | Day120 |
| I_HRS | RS178 | D0 | Day120 |
| I_HRS | RS180 | D0 | Day120 |
| I_HRS | RS190 | D0 | Day120 |
| I_HRS | RS195 | D0 | Day120 |
| I_HRS | RS198 | D0 | Day120 |
| I_ETP | R1003 | Week0 | Week12 |
| I_ETP | R1008 | Week0 | Week12 |
| I_ETP | R1010 | Week0 | Week12 |
| I_ETP | R1011 | Week0 | Week12 |
| I_ETP | R1016 | Week0 | Week12 |
| I_ETP | R1017 | Week0 | Week12 |
| I_ETP | R1023 | Week0 | Week12 |
| I_ETP | R1029 | Week0 | Week12 |
| I_ETP | R1030 | Week0 | Week12 |
| I_ETP | R1031 | Week0 | Week12 |
| I_ETP | R1032 | Week0 | Week12 |
| I_ETP | R1033 | Week0 | Week12 |
| I_ETP | R1034 | Week0 | Week12 |
| I_ETP | R1035 | Week0 | Week12 |
| I_ETP | R1038 | Week0 | Week12 |
| I_ETP | R1041 | Week0 | Week12 |
| I_ETP | R1046 | Week0 | Week12 |
| I_ETP | R1051 | Week0 | Week12 |
| I_ETP | R1055 | Week0 | Week12 |
| I_ETP | R1060 | Week0 | Week12 |

**Table S8. Related to Figure 4.** Count of each category among discovery and validation cohorts

| Count | Baseline | I_MWP | I_LCD | I_HFD | I_HRS | I_ETP | V_IBD-AID | V_WGD |
| --- | --- | --- | --- | --- | --- | --- | --- | --- |
| Responder | 28 | 10 | 7 | 43 | 15 | 3 | 6 | 14 |
| Partial-Responder | 56 | 4 | 3 | 20 | 9 | 4 | 3 | 8 |
| Non-Responder | 584 | 0 | 0 | 8 | 26 | 13 | 6 | 25 |
